# Expression of sphingosine kinase 1 in amoeboid microglial cells in the corpus callosum of postnatal rats

**DOI:** 10.1186/1742-2094-8-13

**Published:** 2011-02-11

**Authors:** Haiyan Lin, Nimmi Baby, Jia Lu, Charanjit Kaur, Chuansen Zhang, Jiajun Xu, Eng-Ang Ling, S Thameem Dheen

**Affiliations:** 1 Department of Anatomy, Second Military Medical University, 800 Xiangyin Road, Shanghai, 200433, PR China; 2 Department of Anatomy, Yong Loo Lin School of Medicine, National University of Singapore, Blk MD10, 4 Medical Drive, 117597, Singapore

## Abstract

Sphingosine kinase 1 (SphK1), a key enzyme responsible for phosphorylating sphingosine into sphingosine-1-phosphate (S1P) has been shown to be expressed in monocytes and monocyte-derived peripheral macrophages. This study demonstrates SphK1 immunoexpression in amoeboid microglial cells (AMC), a nascent monocyte-derived brain macrophage in the corpus callosum of developing rat brain. SphK1 immunofluorescence expression, which appeared to be weak in AMC in normal brain, was markedly induced by lipopolysaccharide (LPS) or hypoxia treatment. Western blot analysis also showed increased expression level of SphK1 in the corpus callosum rich in AMC after LPS treatment. Detection of SphK1 mRNA and its upregulation after LPS treatment was confirmed in primary culture AMC by RT-PCR. Administration of N, N-dimethylsphingosine (DMS), a specific inhibitor of SphK1, effectively reduced upregulated SphK1 immunoexpression in AMC both *in vivo *and *in vitro*. This was corroborated by western blot which showed a decrease in SphK1 protein level of callosal tissue with DMS pretreatment. Remarkably, LPS-induced upregulation of the transcription factor NFκB was suppressed by DMS. We conclude that SphK1 expression in AMC may be linked to regulation of proinflammatory cytokines *via *an NFκB signaling pathway.

## Background

Sphingosine kinase (SphK) is an enzyme that phosphorylates sphingosine to sphingosine-1-phosphate (S1P). SphK has two isoforms, SphK1 and SphK2, that have different properties and subcellular localizations [1,2]. While much has been reported on the expression and roles of SphK1 in different cells and its participation in distinct biological functions [1], the biological functions of SphK2 are not yet clearly defined. This study focused on SphK1 in view of its potential role in the central nervous system (CNS) [1]. SphK1 is mainly and abundantly expressed in cytosol of hippocampal neurons, endothelial cells, cerebellar granule cells and astrocytes of rat brain; and in cultured oligodendrocytes and murine BV2 cells [3-7]. SphK1 is also expressed in other tissues including heart, lungs, and kidneys [8,9]. SphK1 overexpression enhances cell survival and cell proliferation [10]. Studies of expression and functions of SphK1 in several types of human cancer tissues [11,12], primary human astrocytes [13], various glioblastoma cell lines [14,15], and cultured brain endothelial cells [6] have shown that it may serve as a novel and useful prognostic marker for astrocytoma and, furthermore, it may play an important role during the development and progression of neoplastic diseases [16,17]. Despite this, however, there is only a modicum of information on the role of SphK1 in the brain, especially in regard to its localization in microglia *in vivo*. This is particularly so in amoeboid microglial cells (AMC) in the developing brain, which are considered to be the nascent brain macrophages [18]. Indeed, as far as can be ascertained, expression of S1P receptors has been reported in microglia only in culture [5,19]. In this connection, it is relevant to note that SphK 1 is highly expressed in blood monocytes [20], the precuror cells of AMC [18].

We have reported recently that AMC, when challenged with LPS or exposed to hypoxia, release large amounts of inflammatory cytokines including TNF-α and IL-1β [21]. The production of TNF-α and IL-1β involves SphK1 in LPS-activated monocytes/macrophages and BV2 cells [5,22]. Interestingly, SphK1 is linked to TNF-α triggered release of cytokines such as IL-1β and IL-6 [20], indicating its role in proinflammatory activities. Additionally, it has been reported that SphK1 expression in human endothelial cells and U87MG glioma cells may also be regulated under hypoxic condition [23,24]. In the light of the above, this study sought to determine if AMC in postnatal rats express SphK1 and, if so, to determine how it might be regulated during microglial activation induced by LPS or hypoxia. To this end, blockade or down-regulation of SphK1 by its specific inhibitor namely, N, N-dimethylsphingosine, (DMS), could help to unravel the functions of SphK1 [25]. The information is important as AMC have been shown to be involved in neuroinflammatory processes [26] that have been implicated in the early stages of various neurodegenerative disorders [27]. Hence, ascertaining SphK1 expression in microglia in normal and under altered conditions could lead to a better clarification of its specific role in neuroinflammation.

## Methods

Wistar rats of different age groups (1, 3, 5, 7, 14 and 21 days, and 1 month) were purchased from the Laboratory Animal Centre, National University of Singapore. All experiments were carried out in accordance with the International Guiding Principles for Animal Research, as adopted by the Institutional Animal Care and Use Committee, National University of Singapore. All efforts were made to minimize pain and the number of rats used.

### Normal postnatal rats

Wistar rats aged 1, 3, 5, 7, 14 and 21 days were used for immunofluoresence studies (n = 3, at each age). The rats were anesthetized with Nembutal (sodium pentobarbital, 60 mg/kg) and perfused transcardially with Ringer's solution followed by 2% paraformaldehyde in 0.1 M phosphate buffer. Following perfusion, the brain was removed, post-fixed in the same fixative and cryoprotected in 30% sucrose for 24 h. Frozen sections at 30 μm were cut coronally through the forebrain with a cryostat (Model CM 3050; Leica Instruments GmbH, NUBLOCH, Germany) and mounted onto gelatin-coated slides and stored at -20°C until use.

### Injection of lipopolysaccharide

Fifty four 5-day-old rats were divided into 6 groups. In each group (n = 9), 3 rats were used for immunofluoresence staining and 6 for reverse transcription polymerase chain reactions (RT-PCR) and western blotting. In the experimental groups, the rats were given an intraperitoneal (i.p.) injection of 100 μL lipopolysaccharide (LPS) (1 mg/kg; Cat. No. L2654, Sigma-Aldrich, MO, USA). Three rats each were sacrificed at 30 min, 1 h and 6 h after LPS injection; 9 rats receiving an equal volume of saline served as matching controls. In another experimental group receiving DMS and LPS treatments, the rats were given an i.p. injection of 100 μl DMS (100 μM, Cat. No.310500, Calbiochem, USA) followed by LPS (3 mg/kg) injection 15 min later. For immunostaining, the brain was removed and sectioned, and the tissue sections stored with the same procedure as described above. For western blotting and quantitative RT-PCR analysis, the brain was exposed and the cerebral hemispheres dissected under deep anaesthesia. Fresh tissue samples of the corpus callosum above the lateral ventricles were carefully dissected and kept in liquid nitrogen until further use.

### Hypoxic exposure

Forty five rats, aged 5 days, were divided into 5 groups (n = 9 each). Twenty seven rats were placed in a chamber filled with a gas mixture of 5% oxygen and 95% nitrogen (MCO18M; Sanyo Biomedical Electrical Co Ltd, Tokyo, Japan) for 2 h. They were allowed to recover in air and at room temperature for 15 min, 1 h and 6 h before being sacrificed. In another experimental group, the rats received DMS pretreatment followed by hypoxia exposure 15 min later. Nine rats were given firstly with an i.p. injection of 100 μl DMS (100 μM, Cat. No.310500, Calbiochem, USA), followed by an acute hypoxic exposure in an incubator for 2 h. Nine rats kept outside of the chamber were used as normal control. In each group, 3 rats were used for immunofluorescence staining, with the remaining 6 rats used for RT-PCR and western blotting analysis. Tissue sections and fresh corpus callosum were removed for different methods as described above.

### Immunofluorescence staining

Tissue sections were rinsed in phosphate-buffered saline (PBS) and then incubated in 5% normal goat serum diluted in PBS for 1 h at room temperature. Following removal of serum, tissue sections were incubated overnight with a mixture of primary antibodies containing OX-42 (1:300, mouse monoclonal IgG, Cat. No. CBL1512, Chemicon, USA) and anti-SphK1 (1:100, rabbit polyclonal, Cat. No.sc-48825IgG, Santa Cruz, USA) antibodies at room temperature. Some sections were incubated with preimmune rabbit IgG (Cat. No. 17-615, Millipore, USA) as isotypic controls. Sections from LPS or DMS/LPS treated samples were also incubated with OX-42 along with either anti-tumor necrosis factor-α (TNF-α), anti-interleukin-1β (IL-1β) (TNFα, rabbit polyclonal, Cat. No. AB1837P; IL-1β, rabbit polyclonal, Cat. No. AB1832P, Chemicon, USA) or anti-NFκB (1:200, rabbit polyclonal, sc-109, Santa Cruz) antibodies. On the next day, the sections were washed in PBS-Triton X 100 (TX). They were incubated with a mixture of secondary antibody: FITC-conjugated goat anti-rabbit IgG (1:200, Chemicon, USA) and Cy3-conjugated goat anti-mouse IgG (1:200, Chemicon, USA) for 1 h. After washing in PBS-TX, sections were stained with 4', 6-diamidino-2-phenylindole (DAPI, 1 μg/mL) for 2 min at room temperature. They were then rinsed in PBS-TX and mounted with a fluorescent mounting medium (DAKO Cytomation, Glostrup, Denmark). Colocalization was examined under a confocal microscope (LSM FV1000; Olympus Company Pte. Ltd., Tokyo, Japan).

### Western blot assay

Proteins were extracted from the frozen corpus callosum of saline-, LPS- and DMS+LPS-injected rats using a protein extraction kit (Pierce Biotechnology Inc, IL, USA). The concentration of protein was measured using bovine serum albumin (Sigma-Aldrich, St Louis, MO, USA) as a standard. Samples of supernatant containing 50 μg protein were heated at 95°C for 4 min and separated in 10% sodium dodecyl sulfate-polyacrylamide gels. The proteins were transferred to immobilon polyvinylidene difluoride transfer membranes using a semi-dry electrophoretic transfer cell (Bio-Rad, CA, USA). The membranes were blocked with 5% non-fat dry milk in Tris-buffered saline containing 0.1% Tween 20 (TBST) for 1 h at room temperature. The membranes were incubated with the primary antibody anti-Sphk1 (1:1000, rabbit polyclonal IgG, Cat. No.sc-48825, Santa Cruz, USA) at 4°C overnight with shaking. After thorough washing in Tris buffered saline with Tween-20 (TBST), the membranes were incubated with horseradish peroxidase (HRP)-conjugated secondary antibody (1:5000, goat anti-rabbit IgG, Cat. No. GF9501119, Pierce, USA) for 1 h at room temperature. The immunoproducts were detected using a chemiluminescence detection system according to the manufacturer's instructions (Supersignal West Pico Horseradish Peroxidase Detection Kit, Pierce Biotechnology, IL, USA) and developed on a film. The membranes were washed and stripped with Western Blot Stripping Buffer (Prod #21059, Thermo, USA). They were then incubated with primary antibody anti-β-actin (1:10,000, mouse monoclonal, Sigma-Aldrich, MO, USA) at 4°C overnight with shaking. After membranes were incubated with HRP-conjugated secondary antibody (1:5000, goat anti-mouse IgG, Cat. No. GE9542222, Pierce, USA), immunoproducts of β-actin were developed on a film. The optical density of each protein band was quantified by a scanning densitometer and Quantity One Software, version 4.4.1 (Bio-Rad, CA, USA). Each lane of protein band density was normalized with corresponding β-actin density.

### Real time reverse-transcription polymerase chain reactions (RT-PCR)

Total RNA was extracted from frozen corpus callosum or primary culture microglia using RNeasy Mini kit (Qiagen, Valencia, CA, USA) and its concentration was quantified with a biophotometer (Eppendorf, Westbury, NY, USA). Reverse transcription was carried out as follows: A mixture of 2 μg total RNA and 2 μl Oligo (dT) was heated at 70 °C for 5 min, rapidly cooled down on ice and mixed with reaction mixture containing 5 μl M-MLV RT 5X Buffer (Cat. No M531A, Promega, USA), 0.5 μl deoxyribonucleotide triphosphate (dNTP, 25 μM, Cat No. U1240, Promega, USA), 0.7 μl RNAse inhibitor (2500U, RNasin^® ^Cat. No N211A, Promega, USA) and 1 μl M-MLV reverse transcriptase (10000u; Cat. No M170A, Promega, USA). The reaction was made up to 25 μl with RNA-free water and then incubated at 42 °C for 1 h and at 70 °C for 10 min. cDNA was synthesized and stored at -80°C until its use. RT-PCR was carried out on a LightCycler instrument using QuantiTect SYBR Green PCR Kit (Cat. No. 204141, QIAGEN, USA) according to the manufacturer's instructions. For RT-PCR, reaction mixture containing 5 μl QuantiTect SYBR Green PCR Master Mix, 0.5 μl of 10 μM forward primer, 0.5 μl of 10 μM reverse primer, cDNA template and RNAse-free water was prepared and transferred into capillary glass tubes. PCR was performed under the following conditions: denaturation at 95 °C for 15 min, followed by 45 cycles of denaturation at 94 °C for 15 s, annealing at 61 °C for 22 s, and extension at 72 °C for 30 s.

Expression of target genes was measured in triplicate and was normalized to β- actin, an internal control. The RT-PCR products were subjected to 2% agarose gel electrophoresis, stained with 1mg/mL ethidium bromide, and photographed using ChemiGenius2 image analyzer (SYNGENE, Cambridge, UK). Forward and reverse primer sequences for each gene and their corresponding amplicon sizes are provided in Table 1. The predicted size and single band confirmed the amplicons and that the respective primers were specific. Fold change of target genes' expression was calculated according to the formula: fold change = 2^-[[Ct (control) gene X-Ct (control) actin]-[Ct (activated) gene X-Ct (activated) actin]] ^[28].

**Table 1:** Primer sequences used for RT-PCR

Name	Forward	Reverse	Size (bp)
TNF-α	ccaacaaggaggagaagttcc	ctctgcttggtggtttgctac	134
IL-1β	ggaacccgtgtcttcctaaag	ctgacttggcagaggacaaag	123
SphK1	tcagtctgtcctggggtttc	tcctccagaggaacgaggta	226
			
β- actin	tcatgaagtgacgttgacatccgt	cctagaagcatttgcggtgcaggatg	285

### Primary culture of microglial cells

Mixed glial cells were prepared from the cerebrum in neonatal rats aged 3 days and cultured in a flask containing Dulbecco's modified Eagle's medium (DMEM), 10% fetal bovine serum (FBS), 10 ml/L antibiotic-anti-mycotic (Cat. No. A5955, Sigma, USA), 0.1 mM nonessential amino acid (Cat. No. 11140-050, Invitrogen, USA) and 1 ml/L insulin (Cat. No. I-0516, Sigma, USA). The complete medium was replaced at 24 h and then every 3-4 days. Microglial cells were isolated at 10 days with 0.25% trypsin including 1 mM ethylene diamine tetra acetic acid (EDTA) for 10-20 min at 37 °C[29]. With a complete detachment of upper cell layer, microglial cells remained attached to the bottom of the flask and DMEM containing 10% FBS was added for trypsin inactivation. Microglial cells were cultured in complete medium overnight for their identification. The purity of microglial cells was confirmed by OX-42 labeling.

### Immunofluoresence staining of cultured microglial cells

Microglial cells were plated on poly-L-lysine coated coverslips and cultured in complete medium overnight. The complete medium was then replaced by basic medium and LPS (1 μg/mL) was added into the basic medium for 1 h with or without pretreatment of DMS (15 μM, 15 min). Cells cultured in the basic medium served as the control. The cells were then rinsed with PBS and fixed with 4% paraformaldehyde in phosphate buffer for 30 min at room temperature. Following incubation in normal goat serum, all coverslips with adherent cells were incubated in a mixture of antibodies containing anti-SphK1 (1:100, Santa Cruz, USA) and OX-42 (1:200, Chemicon, USA) at 4°C overnight. The coverslips were washed and incubated with Cy3-conjugated goat anti-rabbit IgG (1:100, Chemicon, USA) and FITC-conjugated goat anti-mouse IgG (1:100, Chemicon, USA) for 1 h at room temperature. Cell nuclei were counterstained with DAPI (1 μg/mL) for 2 min. The coverslips were then washed and mounted with a fluorescent mounting medium (DAKO Cytomation, Glostrup, Denmark). All images were captured under a confocal microscope (LSM FV1000; Olympus Company Pte. Ltd., Tokyo, Japan).

### Quantitative immunofluorescence analysis of microglial cells

Immunofluorescence staining of SphK1, IL-1β, TNF-α and NFκB in microglial cells was digitally captured by using a confocal microscope, and the average intensity of immunofluorescence in microglial cells was estimated using a software system (Fluoview, FV1000). Background (nonspecific extracellular signal) was optimized. These settings, used to optimize image quality, were then applied as standards for the processing of all cell images. Between seven and ten intact microglial cells (showing cellular and nuclear integrity) from each slide were selected and specified by drawing outlines, and their images were acquired in a single channel (showing single colour). The mean value of the intensity in the specified region was measured and normalized with the intensity value obtained from isotypic control sections. Fold changes were calculated and presented in a histogram. The total number of microglial cells on each slide was manually counted in a specified unit area. The percentages of SphK1-, IL-1β-, TNF-α-, and NFκB-positive cells were also calculated by counting the number of positive cells in OX-42-stained microglia.

## Results

### SphK1 immunoexpression in amoeboid microglial cells varies with age in normal rats

**Figure 1:**
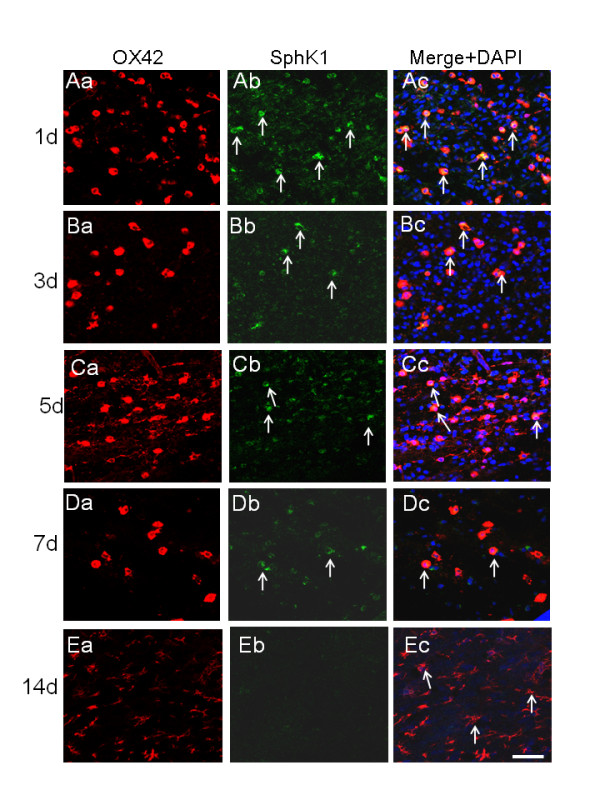
**Confocal images showing SphK1 immunoexpression in OX-42 positive amoeboid microglial cells in different age groups.** Coronal sections through the forebrain of rats aged 1, 3, 5, 7 and 14 d, showing OX-42 (Aa-Ea) and SphK1 (Ab-Eb) expression in amoeboid microglial cells (AMC) in the corpus callosum. SphK1 immunofluorescence is detected in AMC (Ab, Bb, Cb, Db) as confirmed by OX-42 labeling (Aa, Ba, Ca, Da). Co-expression of OX-42 and SphK1 is seen in merged images in Ac, Bc, Cc and Dc (yellow, white arrows). SphK1 immunofluorescence is evident in AMC in rats aged 1, 3 and 5 d but appears to be weaker at age 7 d; it is absent in 14-d-old rats (Ea-c). Nuclei are stained with DAPI. Scale bar = 50 μm.

**Figure 2:**
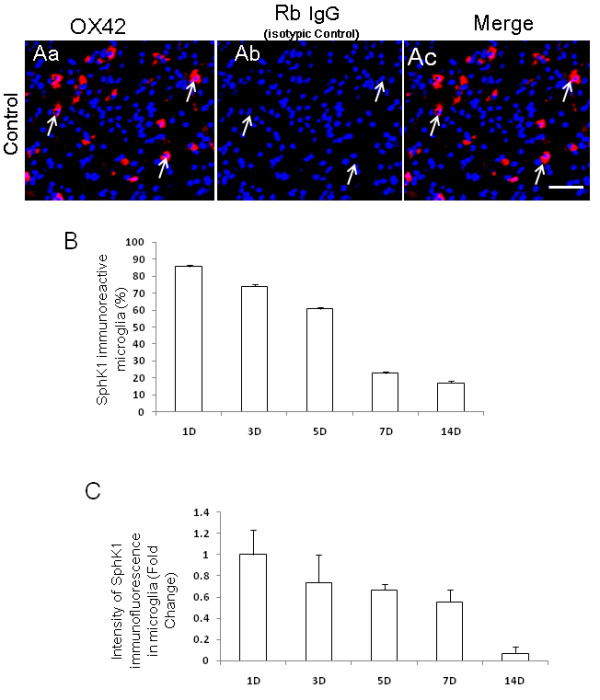
**Isotypic control for SphK1 immunoreactivity.** Sections incubated with rabbit IgG as an isotypic control for anti-SphK1 do not show any immunoreactivity, confirming the specificity of SphK1 immunoreactivity (Aa-c). Nuclei of all cells are stained with DAPI. Quantitative analysis shows that the frequency of SphK1-positive AMC decreases progressively with advancing age. The majority of AMC in 1-5-d-old rats are SphK1 positive. The number of SphK1-positive cells decreased by age 7-14 d (B). Likewise, SphK1 immunofluorescence intensity is attenuated with advancing age (C). Scale bar = 50 μm

AMC in the corpus callosum at different age groups was found to show moderate but definite expression of SphK1 protein as revealed by immunofluorescence staining (Figure 1Aa-Dc, Figure 2B, C). The identification of SphK1-immunoreactive AMC was confirmed by their colocalization with OX42 immunofluorescence. Sections incubated with rabbit IgG as the isotypic control for anti-SphK1 did not show any immunoreactivity, confirming the specificity of SphK1 immunoreactivity (Figure 2Aa-c). The intensity of SphK1 expression in AMC was most intense in corpus callosum of 1- to **5-**day-old rats compared to that of those aged 7 d (Figure 1Da-c, Figure 2C). In rats aged 14 d (Figure 1Ea-c, Figure 2C) and 21 d (data not shown), in which microglia had assumed a ramified shape, SphK1 immunofluorescence was undetectable. Quantitative analysis revealed that the number of SphK1-positive AMC was more numerous in corpus callosum of 1-5 d rats than that of 7-14 d rats (Figure 2B).

### LPS and hypoxia upregulate SphK1 expression in AMC

**Figure 3:**
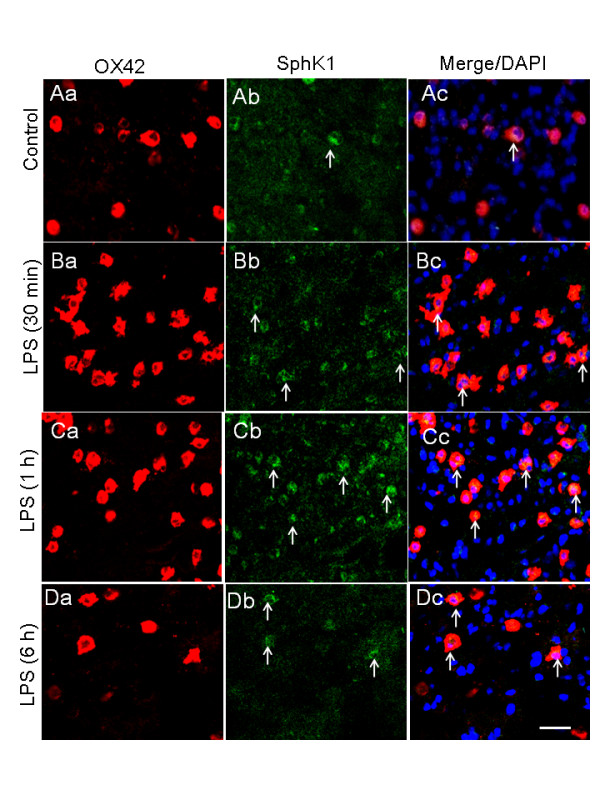
**SphK1 immunoexpression (Ab, Bb, Cb, Db) and OX-42 immunoexpression (Aa, Ba, Ca, Da) in AMC in the corpus callosum of 5-d-old rats given saline and LPS injection and sacrificed at 30 min, 1 h and 6 h**. SphK1 (Bb, Cb, Db) immunoexpression is increased after LPS injection compared with rats given saline injection (Aa - Ac). Panels Ac, Bc, Cc, Dc show co-expression of OX-42 and SphK1 in AMC (arrows) stained with nuclear DAPI. Scale bar = 50 μm

**Figure 4:**
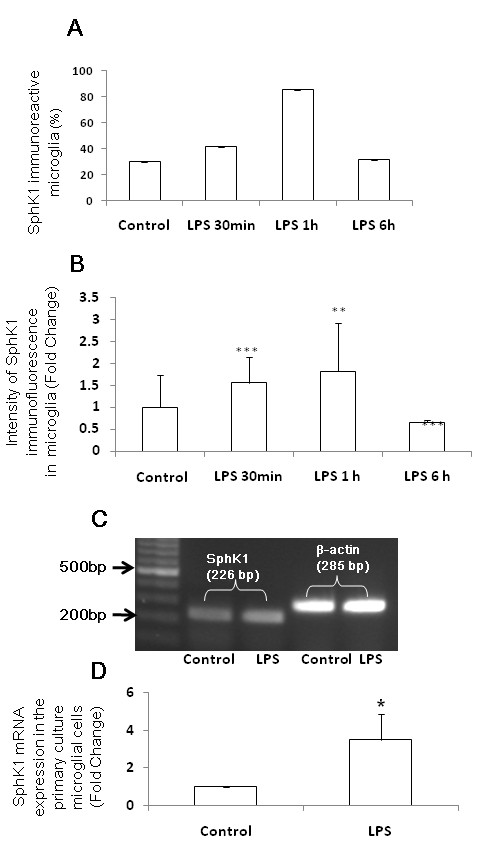
**Quantitative analysis of number, intensity and mRNA expression of SphK1 positive AMC.  **The number of SphK1-positive AMC (co-expressing OX-42) is increased 1 h after LPS injection (A). The intensity of SphK1 immunofluorescence is also increased significantly at 30 min to 1 h compared with that 6 h after LPS injection and that of control (B). SphK1 mRNA expression (C) is detected in primary microglial cells *in vitro *and its expression was induced significantly in cells exposed to LPS for 1 h, as revealed by RT-PCR (D) The data are normalized with the housekeeping gene, β-actin (C) and presented as mean ± S.E. Control *vs *LPS, * *P *< 0.05, ** *P *< 0.01.,*** P < 0.001

In 5-d-old rats injected with LPS, the incidence of SphK1-immunopositive AMC as well as SphK1 immunoreactivity in corpus callosum was increased compared to that of corresponding control rats with saline injection (Figure 3Aa-Dc, Figure 4A, B). The number of SphK1-positive AMC (co-expressing OX-42) was found to be significantly increased at 1 h compared with that at 30 min or 6 h after LPS injection and that of controls (Figure 3Ba-c, Ca-c, Da-c, Figure 4A). The intensity of SphK1 immunofluorescence was found to be increased significantly at 30 min to 1 h compared with that 6 h after LPS injection and that of controls (Figure 4B). Further, mRNA expression of SphK1 (Figure 4C) was detected in primary microglial cells *in vitro *and its expression was induced significantly in cells exposed to LPS for 1 h (Figure 4D).

**Figure 5:**
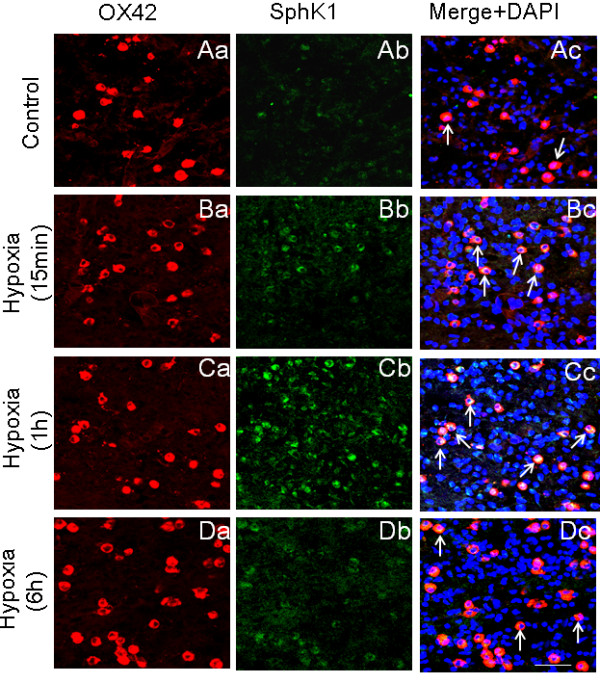
**OX-42 and SphK1 expression in AMC in the corpus callosum of 5-d-old rats subjected to hypoxia for 2 h and sacrificed at 15 min, 1 and 6 h**. OX-42 and SphK1 expression is noticeably increased after hypoxia (Ba-b, Ca-b, Da-b) compared with that in control (Aa-b). Co-expression is seen in merged images in Ac, Bc, Cc and Dc (arrows). Note that SphK1 expression is most intense at 1 h (Cc). Nuclei are stained with DAPI. Scale bar = 50 μm.

**Figure 6:**
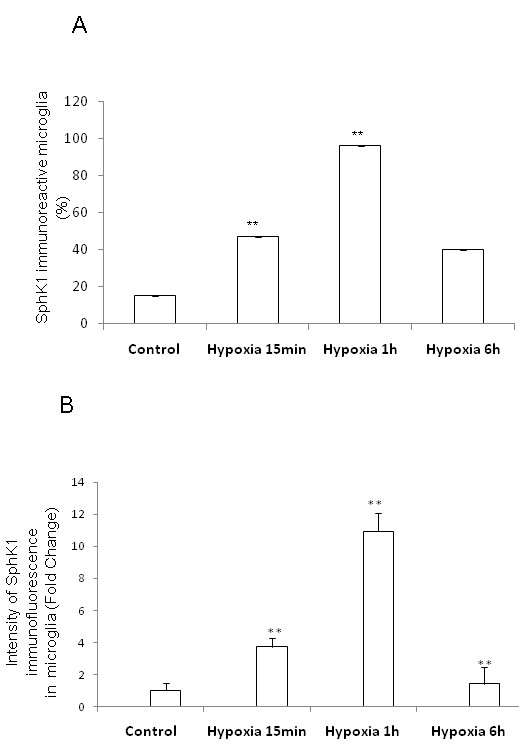
**Quantitative analysis of number and intensity of SphK1 positive AMC.** Significantly increased numbers of SphK1-positive AMC (co-expressing OX-42) are evident in 1 h after hypoxia (A). The intensity of SphK1 immunofluorescence is increased significantly at 15 min and 1 h compared with that 6 h after hypoxia and that of control (B). The data are presented as mean ± S.E. Control *vs *Hypoxia, * *P *< 0.05, ** *P *< 0.01

In rats subjected to hypoxic exposure, SphK1 immunoexpression in AMC was drastically increased (Figure 5A-D, Figure 6A). Virtually all OX-42-positive AMC exhibited more intense SphK1 immunofluorescence 1 h after hypoxia, compared with that in control rats (Figure 5Ca-c, Figure 6A). At 6 h after hypoxia, SphK1 immunofluorescence in AMC appeared to be diminished, although OX-42 immunofluorescence in AMC remained intense and the cells were hypertrophic (Figure 5Da-c).

### DMS inhibits SphK1 expression in amoeboid microglial cells

**Figure 7:**
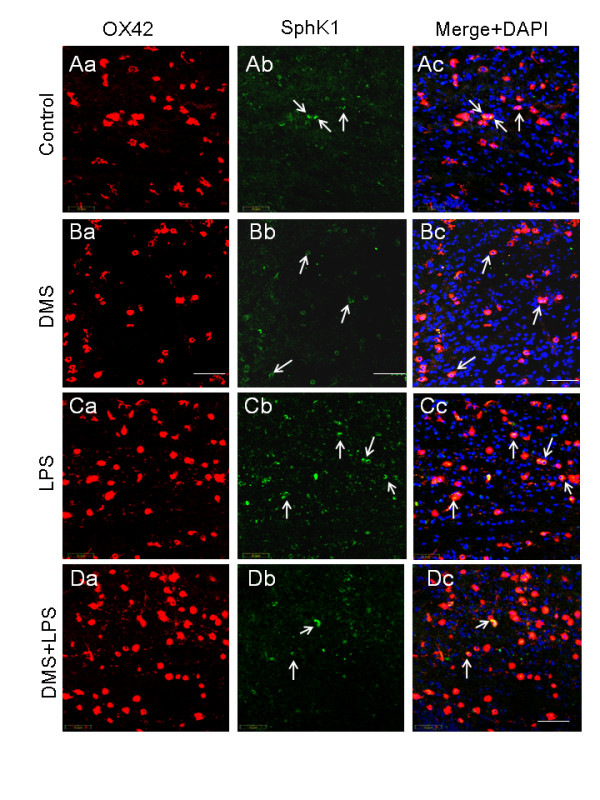
**SphK1 (Ab Bb, Cb, Db) expression in 5-d-old rats receiving saline, DMS, LPS or DMS + LPS injection, and sacrificed 1 h later**. Immunofluorescence analysis reveals colocalization of OX-42 (Aa, Ba, Ca, Da) and SphK1 (Ab, Bb, Cb, Db) in AMC of corpus callosum as shown in the merged images (Ac, Bc, Cc, Dc). The intensity of SphK1 expression in AMC is clearly enhanced after LPS injection (Cb) when compared with that of the control (Ab) and DMS (Bb) groups (**arrows**). In rats treated with either DMS alone or DMS + LPS, SphK1 immunofluorescence intensity in AMC appears to be unchanged (Bb, Db). Nuclei are stained with DAPI. Scale bar = 50 μm.

**Figure 8:**
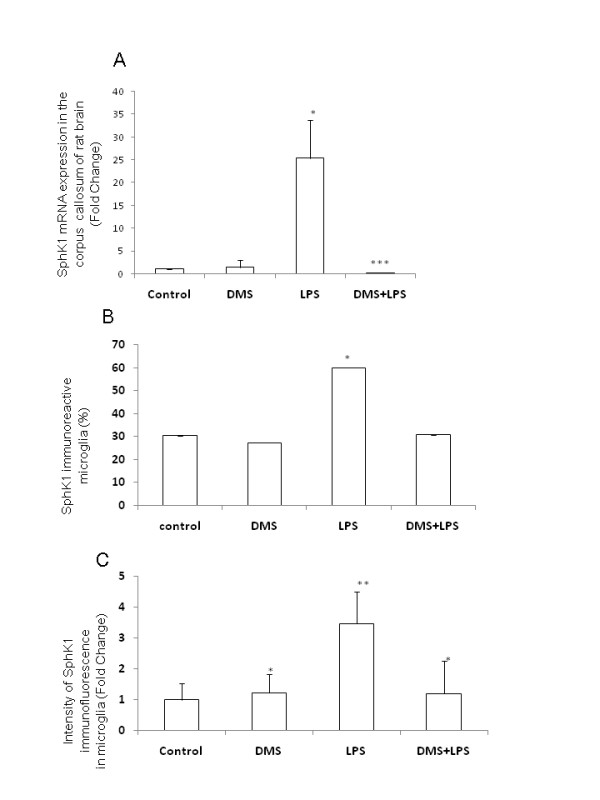
**Levels of SphK1 mRNA expression and immunofluorescence intensity and  the number of SphK1 positive cells are reduced after LPS and DMS treatments.** RT-PCR analysis demonstrates that SphK1 mRNA expression is increased more than 25 fold in corpus callosum of rats injected with LPS, and the concomitant injection of DMS prevented this increase of SphK1 (A). Injection of DMS alone did not have any effect on SphK1 mRNA expression. Quantitative analysis shows that the number of SphK1-positive AMC decreased in rats injected with DMS+LPS, when compared to that in rats injected with LPS alone (B), Further, the intensity of SphK1 immunofluorescence was also decreased in rats injected with DMS+LPS when compared to that in rats injected with LPS alone (C). Data are presented as mean ± S.E. * *vs *control, * *P *< 0.05, ** *P *< 0.01, *** P < 0.001 Scale bar = 50 μm.

**Figure 9:**
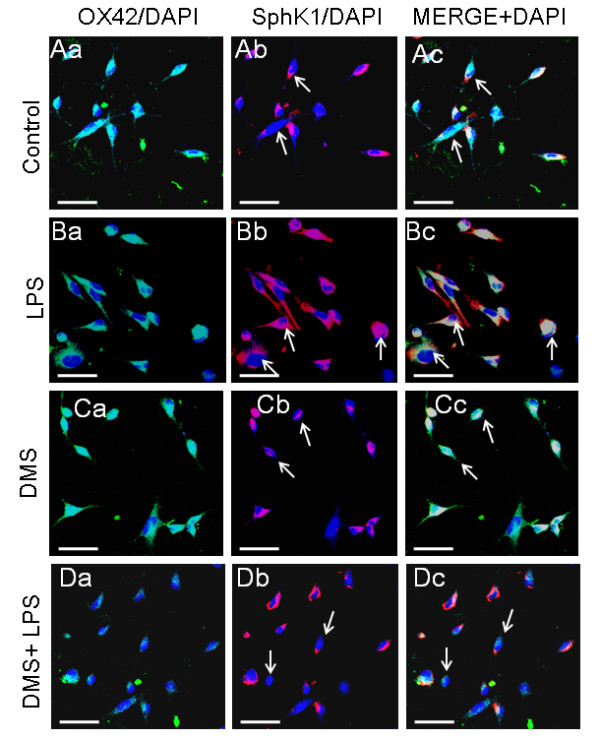
**SphK1 immunoexpression in AMC after LPS and DMS treatments (Aa-c).** The cells treated with LPS (Ba-c) appear hypertrophic (arrows) and exhibit enhanced SphK1 immunoexpression (Bb) as evident in the merged image (Bc). In cells treated with DMS alone or with DMS + LPS (Ca-c, Da-c), the intensity of SphK1 expression appears to be unaltered, compared to that of control (Aa-c). Nuclei are stained with DAPI. Scale bar = 50 μm.

**Figure 10:**
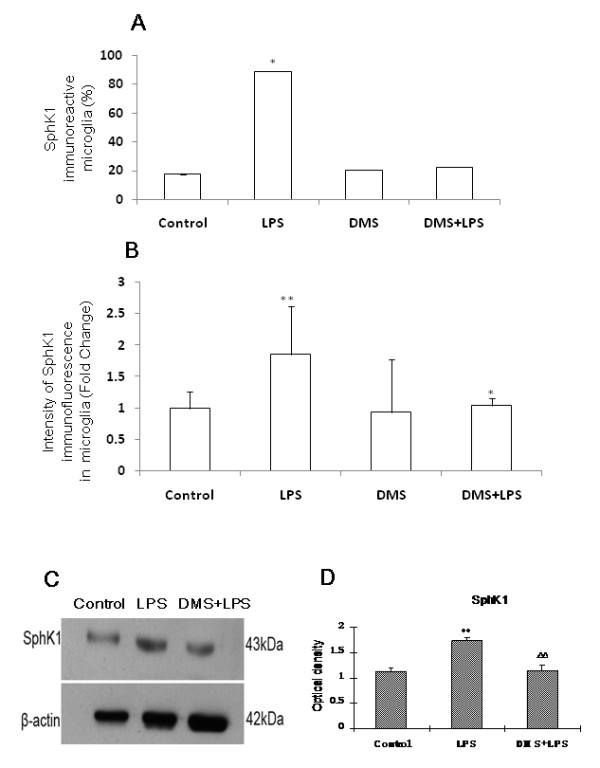
**Number, intensity and protein expression in SphK1 positive cells after LPS and DMS treatments.** Quantitative analysis shows that the number of SphK1-positive AMC increased in cultures treated with LPS when compared to that in untreated cultures (A). This increase in number was not evident in cultures treated with DMS alone or DMS+LPS. Quantitative analysis reveals that the intensity of SphK1 immunofluorescence is increased in cultures treated with LPS when compared to that of untreated cultures (B). Western blot analysis of SphK1 protein levels in the corpus callosum of rats 1 h after LPS or DMS+LPS injection, and corresponding saline controls has been carried out (C, D). C, SphK1 (43kDa) and β-actin (43kDa) immunoreactive bands are shown. D, The optical density of SphK1 following LPS treatment is significantly increased when compared with the corresponding controls. Note that concomitant injection of DMS with LPS did not increase SphK1 protein levels (** *p *< 0.01, ^ΔΔ ^*p *< 0.01). All experiments were carried out in triplicate.

**Figure 11:**
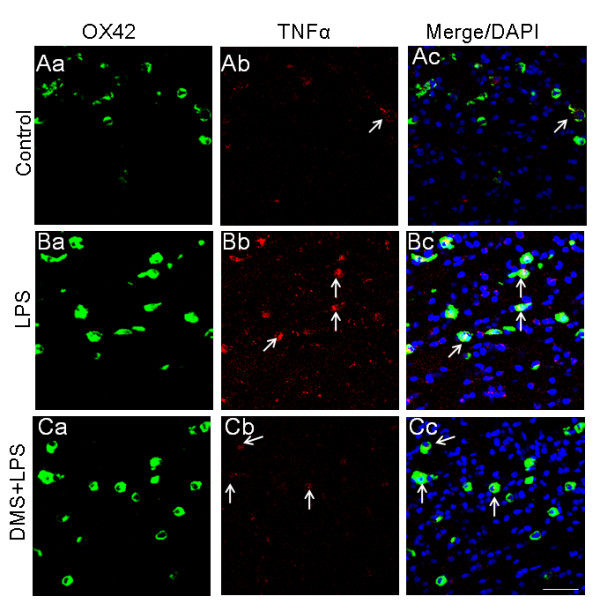
**OX-42 (Aa, Ba, Ca) and TNF-α (Ab, Bb, Cb) expression in 5-d-old rats receiving saline, LPS or DMS + LPS injection and sacrificed 1 h later**. The intensity of TNF-α immunoexpression in AMC is evidently augmented after LPS injection (Bb) when compared with that in the saline group (Ab) (arrows). This is more evident in merged images in Bc in comparison with Ac. In rats treated with DMS + LPS (Ca-c), TNF-α expression appears to be significantly reduced as shown by its co-localization with OX-42 in AMC (Ca-c). Nuclei are stained with DAPI. Scale bar = 50 μm.

**Figure 12:**
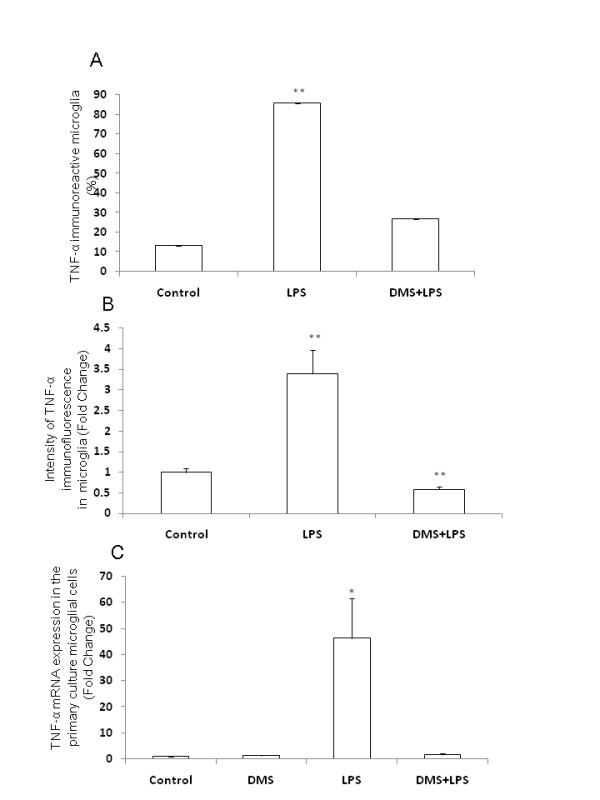
**Number, intensity and mRNA expression in TNF-α-positive AMC after different treatments.** Quantitative analysis shows that the number of TNF-α-positive AMC decreased in rats injected with DMS+LPS when compared to that in rats injected with LPS alone (A). The intensity of TNF-α immunofluorescence is decreased in rats injected with DMS+LPS when compared to that in rats injected with LPS alone (B). RT-PCR analysis demonstrates that TNF-α mRNA expression is increased about 50 fold in primary microglial cells *in vitro *treated with LPS, and that concomitant treatment with DMS prevented this increase in TNF-α (C). Data are presented as mean ± S.E, Control *vs *LPS, **P < 0.01.

**Figure 13:**
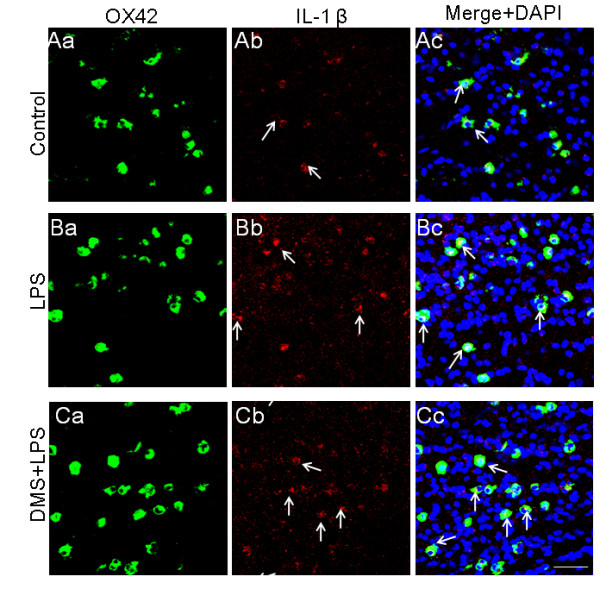
**OX-42 (Aa, Ba, Ca) and IL-1β (Ab, Bb, Cb) expression in 5-d-old rats receiving saline, LPS or DMS + LPS injections and sacrificed 1 h later**. The intensity of IL-1β immunoexpression in AMC is significantly enhanced in LPS-injected rats (Ba-c) when compared with control rats (Aa). Upregulated IL-1β immunoexpression in AMC appears to be sustained in DMS + LPS-injected rats (Ca). Co-expression of OX-42 and IL-1β is evident in DMS + LPS rats as seen in merged images in Cc (arrows). Nuclei are stained with DAPI. Scale bar = 50 μm.

**Figure 14:**
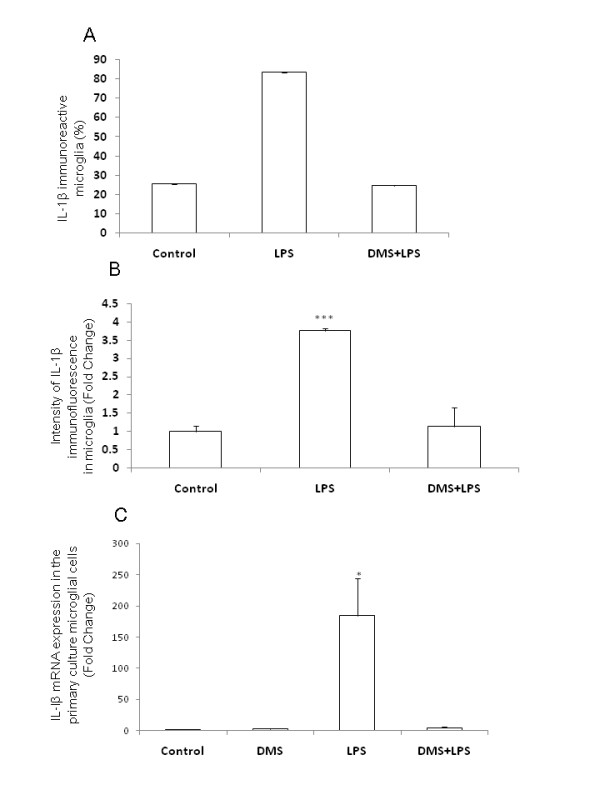
**Number, intensity and mRNA expression in IL-1β-positive AMC after different treatments.** Quantitative analysis shows that there is a significant reduction in the number of IL-1β-positive AMC in rats injected with DMS+LPS when compared to that in rats injected with LPS alone (A). Further, the intensity of IL-1β immunofluorescence is decreased in rats injected with DMS+LPS when compared to that in rats injected with LPS alone (B). RT-PCR analysis demonstrates that IL-1β mRNA expression is increased significantly in primary microglial cells *in vitro *treated with LPS, and that concomitant treatment with DMS prevented this increase in IL-1β (C). Data are presented as mean ± S.E, Control *vs *LPS, ** *P *< 0.01

**Figure 15:**
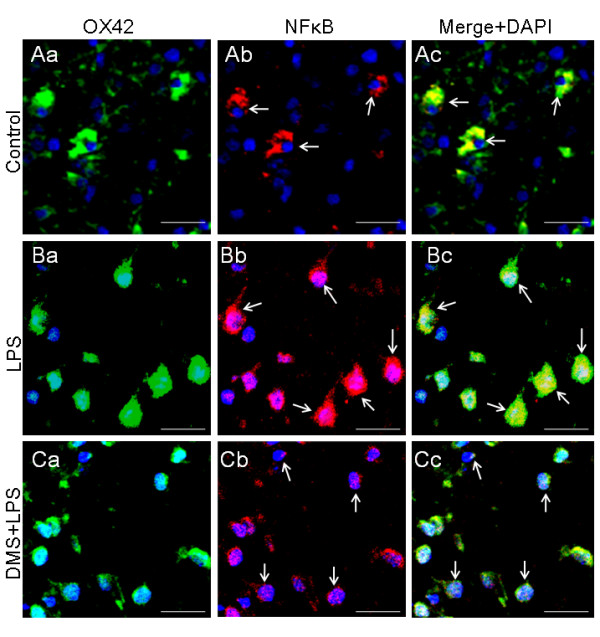
**OX-42 (Aa, Ba, Ca) and NFkB (Ab, Bb, Cb) expression in 5-d-old rats receiving saline, LPS or DMS + LPS injection and sacrificed 1 h later**. NFkB immunoexpression in AMC found in corpus callosum is clearly enhanced in LPS-injected rats (Ba-c) when compared with control rats (Aa-c). Decreased NFkB immunoexpression in AMC is observed in DMS + LPS-injected rats (Ca-c). Co-expression of OX-42 and NFkB is evident in merged images in Ac, Bc and Cc (arrows). Moreover, nuclear translocation of NFκB appears to be more evident and significantly increased in AMC of LPS-injected rats (Bb) when compared with that of control rats or rats injected with LPS+DMS (Fig. Ab, Cb). Nuclei are stained with DAPI. Scale bar = 20 μm.

**Figure 16:**
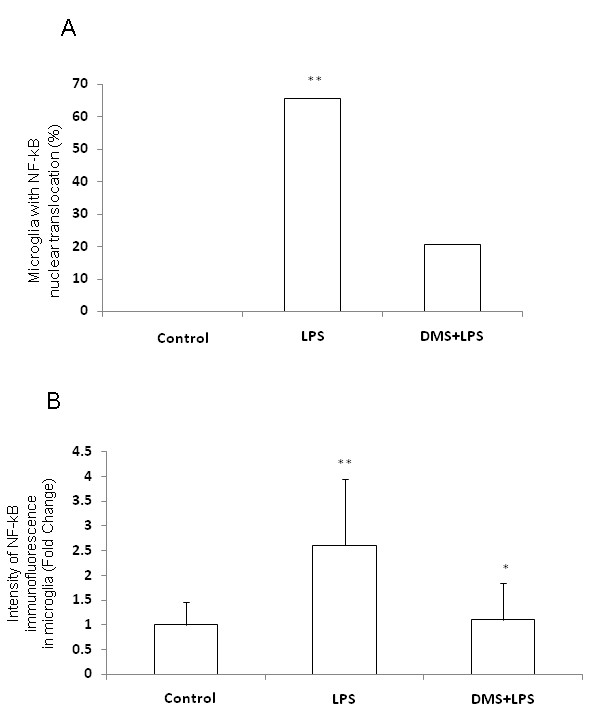
**Quantitative analysis shows that the nuclear translocation and intensity of NFκB immunostaining is significantly increased in AMC of LPS-injected rats when compared with that of control rats or rats injected with LPS+DMS (A,B)**. Data are presented as mean ± S.E, Control *vs *LPS, * *P *< 0.05.

**Figure 17:**
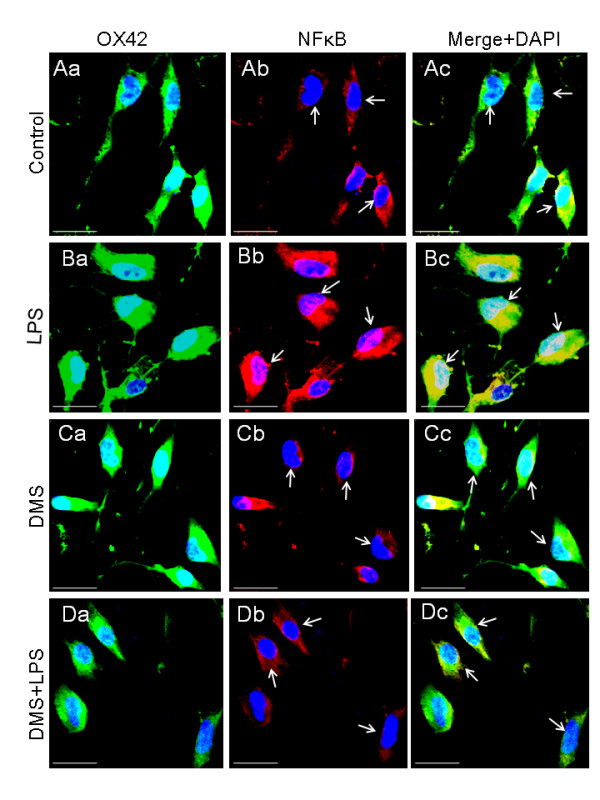
**NFκB expression in primary cultures of AMC labeled by OX-42**. NFkB immunoexpression is clearly enhanced in primary culture microglia exposed to LPS (Ba-c) when compared with control AMC (Aa-c). Decreased NFkB immunoexpression in AMC is observed in AMC treated with either DMS alone (Ca-c) or DMS + LPS (Da-c). Co-expression of OX-42 and NFkB is evident in cells as seen in merged images (arrows).Moreover the nuclear translocation of NFκB appears to be more evident in LPS-treated cells compared with control and DMS + LPS treated cells. Nuclei are stained with DAPI. Scale bar = 20 μm.

As mentioned previously, LPS upregulated SphK1 mRNA and protein expression in AMC of corpus callosum (Figure 7). However, injection of DMS prior to LPS evidently suppressed LPS-induced SphK1 expression in AMC as manifested by its reduced immunofluorescence staining and mRNA expression (Figure 7A-D, Figure 8A-C). A similar phenomenon was observed in microglial cell cultures treated with LPS and DMS + LPS when compared with corresponding controls (Figure 9A-D, Figure 10A, B). The suppression of SphK1 by DMS pretreatment was corroborated by western blot analysis (Figure 10C,D) and RT-PCR (Figure 8A). DMS alone had no significant effect on the expression of SphK1 (Figure 7B, 9C) and this was confirmed by RT-PCR (Figure 8A). LPS-induced TNF-α immunofluorescence intensity (Figure 11C, Figure 12A,B) was found to be reduced in AMC after DMS pretreatment and there was no marked change in LPS-induced IL-1β (Figure 13C, Figure 14A,B) immunofluorescence intensity in those cells. However, real time RT-PCR analysis showed that LPS-induced TNF-α and IL-1β mRNA expression was suppressed in primary culture microglia treated with DMS prior to LPS (Figure 12C and Figure 14C). On the other hand, the intensity of NFκB immunoexpression in AMC found in corpus callosum was significantly increased at 1 h after LPS injection, and NFκB immunofluorescence colocalized with OX-42 (Figure 15Aa-Cc, Figure 16B). Moreover, nuclear translocation of NFκB appeared to be more evident in those cells (Figure 15Bb, Figure 16A). Pretreatment of rats with DMS reduced the incidence of OX-42-labeled, NFκB-immunopositive AMC and NFκB nuclear translocation (Figure 15C). *In vitro *analysis using primary cultures of microglia also showed that LPS treatment induced NFκB translocation from cytoplasm to the nucleus (Figure 17Ba-Bc). This translocation of NFκB was reversed in activated cells pretreated with DMS (Figure 17Da-Dc).

## Discussion

SphK1 is a key enzyme in the sphingolipid metabolic pathway, phosphorylating sphingosine into S1P and regulating intracellular levels of S1P. The SphK1 signaling pathway is involved in cell proliferation, survival, apoptosis, and cell growth arrest as well as regulation of proinflammatory cytokines [30-35]. It has been reported that SphK1 is distributed in rat cerebellum, cerebral cortex, brainstem, striatum and hippocampus; and that SphK1 is a dominant contributor to total SphK activity in mouse brain [36,37]. The present study has shown expression of SphK1 in AMC in corpus callosum of postnatal rat brain *in vivo *as well as in primary microglia *in vitro*. Since SphK1 is constitutively expressed in AMC, it is justified to suggest that this enzyme is linked to normal physiological functions of cells in developing brain. The progressive reduction of SphK1 expression in AMC with advancing age indicates its diminishing role in the developing corpus callosum over a time period wherein it has been reported that all AMC transform at the 14^th ^postnatal day into a more mature form, namely, ramified microglia that have been considered to be the resting form of microglia in the adult brain [18]. It is interesting to note that resting cells typically have very low levels of sphingolipid metabolities, including SphK1 [38].

It is well documented that microglia have been recognized as gatekeepers of CNS diseases and immunology [39] and that microglial cells activated by LPS, by hypoxia, or by neurodegenerative diseases produce increased amounts of proinflammatory cytokines such as TNF-α and IL-1β in the CNS [40-43]. SphK1 participates in inflammatory responses such as TNF-α secretion [31,44,45] and production of cytokines, and blockade of SphK1 activity suppresses such responses in peripheral macrophages and murine BV2 cells [5,13,20,46]. The present results have shown that SphK1 expression in AMC is greatly enhanced by LPS or hypoxia treatment. It is noteworthy that SphK1 immunoexpression was most intense at 1 h after either LPS or hypoxia treatment indicating its early onset of induction and crucial role in the early stage of inflammation in CNS. This is in accordance with the observation that SphK1 expression is induced by LPS in murine macrophages and BV2 cells [5,35]. It is striking that LPS- or hypoxia-induced SphK1 expression in AMC returned to basal levels, suggesting its reversible and transient nature. That LPS and hypoxia could induce upregulated expression of SphK1 in AMC is further supported by the increased level of SphK1 protein in corpus callosum containing a large number of AMC.

Further, reduced SphK1 expression in AMC was evident in postnatal rats treated with DMS, a pharmacological inhibitor of SphK1. A similar phenomenon was observed in primary microglial cultures when compared with that in matching controls. The swift response and upregulation of SphK1 expression in AMC treated with LPS or hypoxia suggests the involvement of the enzyme in an early stage of inflammation. This is further supported by *in vitro *analysis which showed suppression of LPS-induced TNF-α, and IL-1β mRNA expression in primary culture microglia and in BV2 cells treated with DMS [5]. However, pretreatment of LPS-injected postnatal rats with DMS *in vivo *did not alter the expression of all proinflammatory cytokines. For example, there was no marked change in IL-1β protein expression in AMC of corpus callosum as revealed by immunoflurescence. One possible explanation for this discrepancy may be that the dosage of DMS used might not be potent enough to suppress LPS-induced IL-1β expression *in vivo*. We have also shown here that LPS-induced SphK1 and NFκB immunoexpression in AMC is suppressed by DMS. It has been reported that SphK1 is involved in LPS-induced NFκB activation, and that DMS can block LPS-stimulated NFκB activiation [35]. Since DMS appears to act by suppressing protein kinase C, which upregulates the activity of both isoforms of SphK1 and SphK2 [47,48], further studies using SphK1-null mice may be required to confirm the involvement of SphK1 in microglial activation in neuroinflammation.

Notwithstanding the above, it is unequivocal from the present results that AMC in the developing brain constitutively express SphK1 which can be upregulated by LPS and hypoxic exposure. The expression of S1P_1-3, 5 _receptors in AMC (data not shown) suggests that SphK1-S1P pathway operates in AMC and that it may be linked to local neuroinflammation. An example of the latter is periventricular white matter damage in hypoxia, in which AMC are involved through increased release of TNF-α and IL-1β [21]. On the other hand, the interrelations between SphK1 and the above-mentioned various factors and pathways obviously remain complex and await further elucidation. In view of the fact that DMS reduces SphK1 expression in AMC and BV2 cells [5], and that NFκB expression is SphK1 dependent [35], SphK1 appears to be a potential therapeutic target for ameliorating microglia-mediated neuroinflammation.

## Competing interests

The authors declare that they have no competing interests.

## Authors' contributions

HL and NB contributed equally to all experiments. HL wrote the first draft of the manuscript. EAL helped in the design of the experiments and participated actively in discussion of the project and editorial work of the manuscript. CZ and JX are supervisors of HL and participated in the design and discussion of the project. Both JL and CK participated in the design and discussion of the project. STD was instrumental to the execution of the entire project. He is also the Principal Investigator. All of the authors have read and approved the final version of the manuscript.

## References

[B1] Bryan L Kordula T Spiegel S Milstien S Regulation and functions of sphingosine kinases in the brain Biochim Biophys Acta 2008 1781 459 466 18485923 10.1016/j.bbalip.2008.04.008 PMC2712649

[B2] Hait NC Oskeritzian CA Paugh SW Milstien S Spiegel S Sphingosine kinases, sphingosine 1-phosphate, apoptosis and diseases Biochim Biophys Acta 2006 1758 2016 2026 10.1016/j.bbamem.2006.08.007 16996023

[B3] Anelli V Bassi R Tettamanti G Viani P Riboni L Extracellular release of newly synthesized sphingosine-1-phosphate by cerebellar granule cells and astrocytes J Neurochem 2005 92 1204 1215 10.1111/j.1471-4159.2004.02955.x 15715670

[B4] Kajimoto T Okada T Yu H Goparaju SK Jahangeer S Nakamura S Involvement of sphingosine-1-phosphate in glutamate secretion in hippocampal neurons Mol Cell Biol 2007 27 3429 3440 10.1128/MCB.01465-06 17325039 PMC1899953

[B5] Nayak D Huo Y Kwang WX Pushparaj PN Kumar SD Ling EA Dheen ST Sphingosine kinase 1 regulates the expression of proinflammatory cytokines and nitric oxide in activated microglia Neuroscience 2010 166 132 144 10.1016/j.neuroscience.2009.12.020 20036321

[B6] Pilorget A Demeule M Barakat S Marvaldi J Luis J Beliveau R Modulation of P-glycoprotein function by sphingosine kinase-1 in brain endothelial cells J Neurochem 2007 100 1203 1210 10.1111/j.1471-4159.2006.04295.x 17316399

[B7] Saini HS Coelho RP Goparaju SK Jolly PS Maceyka M Spiegel S Sato-Bigbee C Novel role of sphingosine kinase 1 as a mediator of neurotrophin-3 action in oligodendrocyte progenitors J Neurochem 2005 95 1298 1310 10.1111/j.1471-4159.2005.03451.x 16313513

[B8] Facchinetti MM Leocata Nieto F Marquez MG Sterin-Speziale N Stratification of sphingosine kinase-1 expression and activity in rat kidney Cells Tissues Organs 2008 188 384 392 10.1159/000139770 18552482

[B9] Ikeda Y Ohashi K Shibata R Pimentel DR Kihara S Ouchi N Walsh K Cyclooxygenase-2 induction by adiponectin is regulated by a sphingosine kinase-1 dependent mechanism in cardiac myocytes FEBS Lett 2008 582 1147 1150 10.1016/j.febslet.2008.03.002 18339320 PMC2423200

[B10] Olivera A Kohama T Edsall L Nava V Cuvillier O Poulton S Spiegel S Sphingosine kinase expression increases intracellular sphingosine-1-phosphate and promotes cell growth and survival J Cell Biol 1999 147 545 558 10.1083/jcb.147.3.545 10545499 PMC2151183

[B11] French KJ Schrecengost RS Lee BD Zhuang Y Smith SN Eberly JL Yun JK Smith CD Discovery and evaluation of inhibitors of human sphingosine kinase Cancer Res 2003 63 5962 5969 14522923

[B12] Johnson KR Johnson KY Crellin HG Ogretmen B Boylan AM Harley RA Obeid LM Immunohistochemical distribution of sphingosine kinase 1 in normal and tumor lung tissue J Histochem Cytochem 2005 53 1159 1166 10.1369/jhc.4A6606.2005 15923363

[B13] Lai WQ Goh HH Bao Z Wong WS Melendez AJ Leung BP The role of sphingosine kinase in a murine model of allergic asthma J Immunol 2008 180 4323 4329 18322246 10.4049/jimmunol.180.6.4323

[B14] Paugh BS Bryan L Paugh SW Wilczynska KM Alvarez SM Singh SK Kapitonov D Rokita H Wright S Griswold-Prenner I Interleukin-1 regulates the expression of sphingosine kinase 1 in glioblastoma cells J Biol Chem 2009 284 3408 3417 10.1074/jbc.M807170200 19074142 PMC2635028

[B15] Van Brocklyn JR Jackson CA Pearl DK Kotur MS Snyder PJ Prior TW Sphingosine kinase-1 expression correlates with poor survival of patients with glioblastoma multiforme: roles of sphingosine kinase isoforms in growth of glioblastoma cell lines J Neuropathol Exp Neurol 2005 64 695 705 10.1097/01.jnen.0000175329.59092.2c 16106218

[B16] Le Scolan E Pchejetski D Banno Y Denis N Mayeux P Vainchenker W Levade T Moreau-Gachelin F Overexpression of sphingosine kinase 1 is an oncogenic event in erythroleukemic progression Blood 2005 106 1808 1816 10.1182/blood-2004-12-4832 15890687

[B17] Sobue S Iwasaki T Sugisaki C Nagata K Kikuchi R Murakami M Takagi A Kojima T Banno Y Akao Y Quantitative RT-PCR analysis of sphingolipid metabolic enzymes in acute leukemia and myelodysplastic syndromes Leukemia 2006 20 2042 2046 10.1038/sj.leu.2404386 16990773

[B18] Ling EA Wong WC The origin and nature of ramified and amoeboid microglia: a historical review and current concepts Glia 1993 7 9 18 10.1002/glia.440070105 8423067

[B19] Tham CS Lin FF Rao TS Yu N Webb M Microglial activation state and lysophospholipid acid receptor expression Int J Dev Neurosci 2003 21 431 443 10.1016/j.ijdevneu.2003.09.003 14659994

[B20] Zhi L Leung BP Melendez AJ Sphingosine kinase 1 regulates pro-inflammatory responses triggered by TNFalpha in primary human monocytes J Cell Physiol 2006 208 109 115 10.1002/jcp.20646 16575915

[B21] Deng Y Lu J Sivakumar V Ling EA Kaur C Amoeboid microglia in the periventricular white matter induce oligodendrocyte damage through expression of proinflammatory cytokines via MAP kinase signaling pathway in hypoxic neonatal rats Brain Pathol 2008 18 387 400 10.1111/j.1750-3639.2008.00138.x 18371179 PMC8095524

[B22] Sweet MJ Hume DA Endotoxin signal transduction in macrophages J Leukoc Biol 1996 60 8 26 8699127 10.1002/jlb.60.1.8

[B23] Anelli V Gault CR Cheng AB Obeid LM Sphingosine kinase 1 is up-regulated during hypoxia in U87MG glioma cells. Role of hypoxia-inducible factors 1 and 2 J Biol Chem 2008 283 3365 3375 10.1074/jbc.M708241200 18055454

[B24] Schwalm S Doll F Romer I Bubnova S Pfeilschifter J Huwiler A Sphingosine kinase-1 is a hypoxia-regulated gene that stimulates migration of human endothelial cells Biochem Biophys Res Commun 2008 368 1020 1025 10.1016/j.bbrc.2008.01.132 18261991

[B25] Bonhoure E Pchejetski D Aouali N Morjani H Levade T Kohama T Cuvillier O Overcoming MDR-associated chemoresistance in HL-60 acute myeloid leukemia cells by targeting sphingosine kinase-1 Leukemia 2006 20 95 102 10.1038/sj.leu.2404023 16281067

[B26] Li P Kaur C Lu J Sivakumar V Dheen ST Ling EA Expression of cyclooxygenase-2 and microsomal prostaglandin-E synthase in amoeboid microglial cells in the developing brain and effects of cyclooxygenase-2 neutralization on BV-2 microglial cells J Neurosci Res 2010 88 1577 1594 20025057 10.1002/jnr.22319

[B27] Dheen ST Kaur C Ling EA Microglial activation and its implications in the brain diseases Curr Med Chem 2007 14 1189 1197 10.2174/092986707780597961 17504139

[B28] Livak KJ Schmittgen TD Analysis of relative gene expression data using real-time quantitative PCR and the 2(-Delta Delta C(T)) Method Methods 2001 25 402 408 10.1006/meth.2001.1262 11846609

[B29] Saura J Tusell JM Serratosa J High-yield isolation of murine microglia by mild trypsinization Glia 2003 44 183 189 10.1002/glia.10274 14603460

[B30] Cuvillier O Sphingosine in apoptosis signaling Biochim Biophys Acta 2002 1585 153 162 12531549 10.1016/s1388-1981(02)00336-0

[B31] Hammad SM Crellin HG Wu BX Melton J Anelli V Obeid LM Dual and distinct roles for sphingosine kinase 1 and sphingosine 1 phosphate in the response to inflammatory stimuli in RAW macrophages Prostaglandins Other Lipid Mediat 2008 85 107 114 10.1016/j.prostaglandins.2007.11.002 18166496 PMC2290737

[B32] Maines LW Fitzpatrick LR French KJ Zhuang Y Xia Z Keller SN Upson JJ Smith CD Suppression of ulcerative colitis in mice by orally available inhibitors of sphingosine kinase Dig Dis Sci 2008 53 997 1012 10.1007/s10620-007-0133-6 18058233 PMC2660406

[B33] Melendez AJ Sphingosine kinase signalling in immune cells: potential as novel therapeutic targets Biochim Biophys Acta 2008 1784 66 75 17913601 10.1016/j.bbapap.2007.07.013

[B34] Olivera A Rosenfeldt HM Bektas M Wang F Ishii I Chun J Milstien S Spiegel S Sphingosine kinase type 1 induces G12/13-mediated stress fiber formation, yet promotes growth and survival independent of G protein-coupled receptors J Biol Chem 2003 278 46452 46460 10.1074/jbc.M308749200 12963721

[B35] Wu W Mosteller RD Broek D Sphingosine kinase protects lipopolysaccharide-activated macrophages from apoptosis Mol Cell Biol 2004 24 7359 7369 10.1128/MCB.24.17.7359-7369.2004 15314148 PMC507005

[B36] Blondeau N Lai Y Tyndall S Popolo M Topalkara K Pru JK Zhang L Kim H Liao JK Ding K Waeber C Distribution of sphingosine kinase activity and mRNA in rodent brain J Neurochem 2007 103 509 517 10.1111/j.1471-4159.2007.04755.x 17623044 PMC2639651

[B37] Fukuda Y Kihara A Igarashi Y Distribution of sphingosine kinase activity in mouse tissues: contribution of SPHK1 Biochem Biophys Res Commun 2003 309 155 160 10.1016/S0006-291X(03)01551-1 12943676

[B38] Maceyka M Payne SG Milstien S Spiegel S Sphingosine kinase, sphingosine-1-phosphate, and apoptosis Biochim Biophys Acta 2002 1585 193 201 12531554 10.1016/s1388-1981(02)00341-4

[B39] Tambuyzer BR Ponsaerts P Nouwen EJ Microglia: gatekeepers of central nervous system immunology J Leukoc Biol 2009 85 352 370 10.1189/jlb.0608385 19028958

[B40] Allan SM Rothwell NJ Cytokines and acute neurodegeneration Nat Rev Neurosci 2001 2 734 744 10.1038/35094583 11584311

[B41] Nadeau S Rivest S Endotoxemia prevents the cerebral inflammatory wave induced by intraparenchymal lipopolysaccharide injection: role of glucocorticoids and CD14 J Immunol 2002 169 3370 3381 12218159 10.4049/jimmunol.169.6.3370

[B42] Sivakumar V Ling EA Lu J Kaur C Role of glutamate and its receptors and insulin-like growth factors in hypoxia induced periventricular white matter injury Glia 2010 58 507 523 10.1002/glia.20940 19795501

[B43] Streit WJ Walter SA Pennell NA Reactive microgliosis Prog Neurobiol 1999 57 563 581 10.1016/S0301-0082(98)00069-0 10221782

[B44] Ibrahim FB Pang SJ Melendez AJ Anaphylatoxin signaling in human neutrophils. A key role for sphingosine kinase J Biol Chem 2004 279 44802 44811 10.1074/jbc.M403977200 15302883

[B45] Melendez AJ Ibrahim FB Antisense knockdown of sphingosine kinase 1 in human macrophages inhibits C5a receptor-dependent signal transduction, Ca2+ signals, enzyme release, cytokine production, and chemotaxis J Immunol 2004 173 1596 1603 15265887 10.4049/jimmunol.173.3.1596

[B46] Vlasenko LP Melendez AJ A critical role for sphingosine kinase in anaphylatoxin-induced neutropenia, peritonitis, and cytokine production in vivo J Immunol 2005 174 6456 6461 15879148 10.4049/jimmunol.174.10.6456

[B47] Serrano-Sanchez M Tanfin Z Leiber D Signaling Pathways Involved in Sphingosine Kinase Activation and Sphingosine-1-Phosphate Release in Rat Myometrium in Late Pregnancy: Role in the Induction of Cyclooxygenase 2 Endocrinology 2007 149 4669 4679 10.1210/en.2007-1756 18723875

[B48] Edsall LC Van Brocklyn JR Cuvillier O Kleuser B Spiegel S N,N-Dimethylsphingosine is a potent competitive inhibitor of sphingosine kinase but not of protein kinase C: modulation of cellular levels of sphingosine 1-phosphate and ceramide Biochemistry 1998 37 12892 12898 10.1021/bi980744d 9737868

